# Regulating islet stress responses through CD47 activation

**DOI:** 10.1007/s00125-025-06409-3

**Published:** 2025-03-25

**Authors:** Atharva Kale, Mahmoud Azar, Vanessa Cheng, Harry Robertson, Sally Coulter, Paulomi M. Mehta, Sohel M. Julovi, Ellis Patrick, Kedar Ghimire, Natasha M. Rogers

**Affiliations:** 1 Kidney Injury Group, Centre for Transplant and Renal Research, Westmead Institute for Medical Research, Westmead, NSW Australia; 2 Faculty of Medicine and Health, The University of Sydney, Camperdown, NSW Australia; 3 Renal and Transplantation Medicine, Westmead Hospital, Westmead, NSW Australia; 4 School of Mathematics and Statistics, The University of Sydney, Camperdown, NSW Australia; 5 Sydney Precision Data Science Centre, The University of Sydney, Camperdown, NSW Australia; 6 Charles Perkins Centre, The University of Sydney, Camperdown, NSW Australia

**Keywords:** CD47, Diabetes mellitus, ER stress, Hypoxia, Islet transplantation, Islets, Thrombospondin-1

## Abstract

**Aims/hypothesis:**

Diabetes is a global health burden characterised by incremental beta cell loss. Islet transplantation is a recognised treatment for individuals with type 1 diabetes and hypoglycaemia unawareness but broader application is constrained by limited islet survival and function post-transplantation. The underlying molecular mechanisms that induce beta cell dysfunction and demise remain unclear, and therapeutic agents that protect against cellular loss and maintain insulin secretion are in demand as potential treatment options. CD47 is a cell surface protein implicated in cellular stress responses but its role in beta cell function remains relatively unexplored. We hypothesised that modulating CD47 expression would demonstrate a cytoprotective effect in beta cells.

**Methods:**

We used primary murine islets with/without genetic deletion of CD47, as well as human islets and MIN6 cells subjected to pharmacological disruption of CD47 signalling (siRNA or blocking antibody). Metabolic stress was induced in cells by exposure to hypoxia, hyperglycaemia or thapsigargin, and markers of the unfolded protein response, cell survival and insulin secretory function were assessed. Human pancreases from individuals with and without diabetes were examined for evidence of CD47 signalling.

**Results:**

Expression of CD47 and its high affinity ligand thrombospondin-1 (TSP1) was robustly upregulated by exogenous stressors. Limiting CD47 signalling improved markers of senescence, apoptosis, endoplasmic reticulum stress, unfolded protein response, self-renewal and autophagy, and maintained insulin secretory responses. We also found concurrent upregulated expression of CD47 and senescence markers in the endocrine pancreas of aged donors and those with type 2 diabetes. Both CD47 and TSP1 expression were increased in pancreases of humans with type 1 diabetes, as were plasma levels of TSP1.

**Conclusions/interpretation:**

Our study provides key insights into the essential role of CD47 as a novel regulator of islet dysfunction, regulating cytoprotective responses to stress. CD47 may contribute to beta cell damage during the development of diabetes and failure of islet transplant function. Therefore, limiting CD47 activation may be a potential therapeutic tool in conditions where islet function is inadequate.

**Graphical Abstract:**

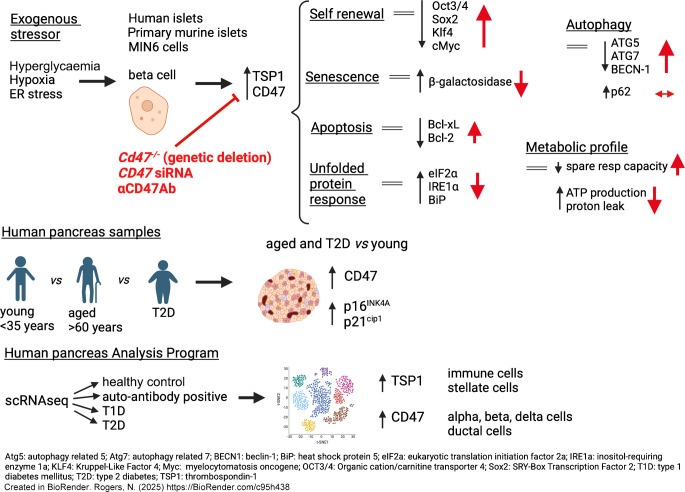

**Supplementary Information:**

The online version contains peer-reviewed but unedited supplementary material available at 10.1007/s00125-025-06409-3.


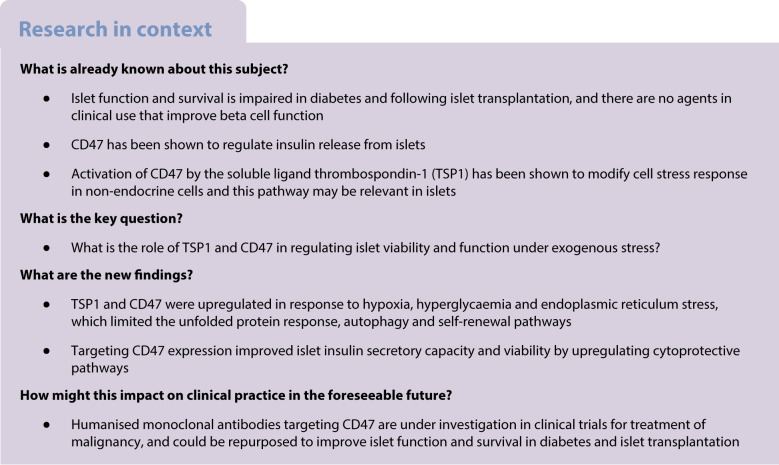


## Introduction

The global burden of diabetes mellitus cannot be underestimated, with the disease affecting >450 million people and being a leading cause of morbidity and mortality due to an accelerated burden of macro- and microvascular complications [1]. All types of diabetes are characterised by beta cell dysfunction and loss leading to critical deficiencies in insulin, and subsequently aberrant metabolic regulation of key energy substrates. Type 1 diabetes is classically defined by immune-mediated destruction of islets but recent evidence suggests that overt hyperglycaemia is preceded by beta cell dysfunction, which pre-dates absolute insulin deficiency. Clinical cohorts reveal limited C-peptide responses [2, 3] and in preclinical studies this phenomenon correlates with endoplasmic reticulum (ER) stress in beta cells. Intact ER function is vital to protein production, folding and packaging (insulin included) and a deterioration leads to activation of the molecular arms of the unfolded protein response (UPR) [4]. In type 2 diabetes, the primary defect may be obesity promoting development of insulin resistance, and synergistic beta cell compensation to ostensibly control hyperglycaemia leads to inflammation and oxidative stress. These pathways converge to impair ER homeostasis, increasing cellular senescence, limiting self-renewal capacity and promoting apoptosis.

Islet transplantation is a recognised treatment for a limited proportion of individuals with type 1 diabetes and hypoglycaemia unawareness. Multiple obstacles prevent its wide application, including the lack of donor organs that limits the initial islet supply to recipients, the stringent purification process that causes mechanical and chemical cellular stress, substantial beta cell destruction in the peri-transplant period due to inflammatory and hypoxic cues in the microenvironment, and chronic attrition due to non-immunological insults that place undue burden on the remaining engrafted islets [5]. Registry data demonstrate satisfactory long-term outcomes following islet transplantation, with 40–50% insulin independence and 95% freedom from severe hypoglycaemic events at 5 years following the last infusion [6]. However, the success of islet transplantation is inferior to whole pancreas transplantation [7]. As outcomes are dependent upon islet number and function, preservation of beta cell viability remains crucial to allograft success.

CD47 is a universally expressed cell surface receptor involved in both the recognition of self-antigen (via signal regulatory protein-α) and cellular responses to stress (via thrombospondin-1 [TSP1]). The latter function of CD47 alters oxidative [8] and ER stress [9], as well as cell death [10], self-renewal [11] and autophagy [12] pathways in non-pancreatic tissues. These mechanisms influence beta cell homeostasis but a cytoprotective function for CD47 in the endocrine pancreas has not been explored. TSP1 is a matricellular protein secreted by nucleated cells and binds with high affinity to CD47 to drive cellular dysfunction. TSP1 is produced by cells in response to hypoxia [13], inflammation [14] and hyperglycaemia [15], which are relevant to the altered microenvironment following intraportal infusion of islets [5], as well as systemic or localised inflammation associated with diabetes. Therefore, activation of CD47 by TSP1 on beta cells is likely to have signalling implications with (patho)physiological relevance.

We recently reported that CD47 plays a crucial role in regulating beta cell function, tonically inhibiting insulin secretion by downstream regulation of the Rho GTPase Cdc42 [16]. In a syngeneic minimal mass islet transplantation model, *Cd47*^−/−^ islets resulted in rapid and sustained glucose control compared with wild-type (WT) (*CD47*^+/+^) cells. We postulated this was due to enhanced insulin secretion; however, we did not investigate the possibility of enhanced survival rate in *CD47*^−/−^ islets contributing to increased mass and improved function. In this study we explore whether modulating CD47 signalling in islets, through genetic deletion, knockdown or targeted antibody blockade, imparts resistance to exogenous stressors that would be manifest during islet isolation and transplantation, as well as in individuals with diabetes in whom there is progressively limited beta cell mass and function.

## Methods

### Animal studies

All animal studies were performed under protocols approved by the Western Sydney Local Health District Animal Ethics Committee (no. 4291 and no. 4369) and in accordance with the Australian code for the care and use of animals for scientific purposes developed by the National Health and Medical Research Council of Australia. *Cd47*^−/−^ mice (B6.129S7-Cd47^tm1Fpl^/J, back-crossed to C57BL/6 mice for 15 generations, https://www.jax.org/strain/003173#) and 3- to 4-month old male C57BL/6 (*Cd47*^+/+^, WT) mice were purchased from Jackson Laboratory (Bar Harbor, ME, USA) and bred at the Australia Bio Resources (Moss Vale, NSW, Australia). For experimental work, male mice were housed in the Westmead Institute for Medical Research Animal Research Facility with ad libitum access to regular chow (8% energy from fat; Gordon’s Specialty Stockfeeds, Yanderra, Australia) and water.

### Human plasma and tissue samples

Healthy individuals or individuals with type 1 diabetes and normal renal function (based on eGFR determined by CKD-EPI) were recruited from outpatient clinics at Westmead Hospital. Blood was collected in K_2_-EDTA tubes without a tourniquet using a 23-gauge needle, and placed immediately on ice. Platelet-poor plasma was generated by centrifugation at 2500 *g* for 15 min at 4°C without brake, then stored at −80°C until analysis. Plasma TSP1 concentration was determined by ELISA (Abcam, Cambridge, UK). The study was approved by the Human Research Ethics Committee of Western Sydney Local Health District (HREC LNR/12/WMEAD/114 and LNRSSA/12/WMEAD/117). All participants provided written consent.

Human pancreas samples used in this study were from deceased donors whose organs were consented for research and unsuitable for either whole-organ or islet transplantation. This study was approved by the Human Research Ethics Committee of Western Sydney Local Health District (HREC 2021/PID02353). All families of organ donors provided written informed consent. Donor details are listed in electronic supplementary material (ESM) Table 1.

### Primary murine islet isolation

Mouse islet isolation was performed as described previously [17] and outlined in detail in ESM Methods. Islets were stained with Dithizone dye (Cole-Parmer Scientific Experts, Vernon Hills, IL, USA) before counting and use in experimental work.

### Human islet isolation

Research-consented human islets unsuitable for clinical transplantation were isolated following the standard protocol carried out by the National Islet Transplantation Consortium at Westmead Hospital [18]. Donor details are provided in the human islet checklist (see ESM).

### Islet exogenous stressor experiments

Murine islets, human islets or MIN6 cells (no. C0018008; AddexBio, San Diego, CA, USA) were exposed to normoxia (fraction of inspired oxygen [F_I_O_2_] 21%) or hypoxia (F_I_O_2_ 1%) for 24 h and then stimulated with basal (2.8 mmol/l) or high (16.7 mmol/l) glucose for 1 h. In separate experiments, cells were supplied with high-glucose media and treated with 0.5 µmol/l thapsigargin (Sigma-Aldrich) for 18 h. The MIN6 cell line (passages 22–30) was tested monthly for mycoplasma and no contamination was identified.

### Limiting CD47 signalling via RNA interference or antibody blockade

Specific to these studies, mouse *Cd47* and control siRNA (Ambion, Life Technologies, Austin, TX, USA) and human *CD47* and control siRNA (ThermoFisher Scientific, Waltham, MA, USA) were used as published previously [16]. In brief, MIN6 cells or human islets were seeded onto 6 cm petri dishes, serum-starved for 1 h, then were treated with either *CD47*-targeted or scrambled control siRNA according to the manufacturer’s instructions (Ambion, Protocol Pub. No. MAN0007836 Rev. 1.0) for 48 h. For antibody blockade, cells were supplied with DMEM high-glucose media and treated with IgG isotype control or anti-CD47 antibody (1 μg/ml) for 15 min prior to experiment initiation.

### Insulin secretion measurements

Following respective cell culture experiments, supernatant fractions were collected and any residual cells were removed from the media by centrifugation at 200 *g* for 4 min at 4°C. Samples were frozen at −80°C until insulin secretion was determined in bulk by ELISA according to the manufacturer’s instructions (Ultra-sensitive Mouse Insulin ELISA no. 90080; Crystal Chem, Elk Grove Village, IL, USA).

### Cell viability, senescence and LDH assays

Cell viability was measured using an XTT Cell Viability Kit (no. 9095, Cell Signalling Technology). Briefly, 10^4^ WT or *CD47*^−/−^ islets/well were seeded into a 96-well microplate and viability was determined at 450 nm using Proteomics SpectraMax iD5 Plate Reader (VWR International, Radnor, PA, USA).

Senescence was assessed with a Mammalian β-Galactosidase Assay Kit (no. 9860, ThermoFisher Scientific). MIN6 cells were treated with control scramble or *CD47* siRNA according to the protocol previously described [16]. Cells were then exposed to hypoxia, supplied with β-galactosidase staining solution and incubated at 37°C overnight. Assessment was performed under light microscopy, and the per cent area of blue dye staining from at least five areas per well per group were evaluated using ImageJ (https://imagej.net/ij/; last accessed 18 Dec 2024).

Lactate dehydrogenase (LDH) from cell culture supernatant fractions was measured using the Lactate Dehydrogenase Assay Kit (ab102526, Abcam) according to the manufacturer’s instructions. In brief, supernatant fractions were stored at −80°C, thawed simultaneously and brought to room temperature. The NADH standard, dilutions and samples were run in duplicate, and the plate was read initially at 450 nm (Proteomics SpectraMax iD5 Plate Reader). Absorbance was read a second time after incubating the plate for 30 min at 37°C, and this reading was subtracted from the initial reading. LDH activity (mU/ml) was determined via the formula: (B × sample dilution) / (30 min × pre-treated sample volume), where B is the difference in absorbance reading. Corrected absorbance values were calculated to generate a standard curve and LDH concentration in samples was determined using the formulaic calculation provided.

### Seahorse assay

To assess metabolic responses in cells, Agilent Seahorse XF Cell Mito Stress Test and XFe24 Extracellular Flux Assay Kit (Agilent Technologies, Santa Clara, CA, USA) assays were used. MIN6 cells were seeded at 10^5^ cells/well in a Seahorse assay plate and treated with *CD47* siRNA for 48 h. A day prior to running the assay, a sensor cartridge plate was hydrated by supplying Seahorse calibrant to each well and incubating it overnight in a non-CO_2_ incubator. On the day of the assay, compounds were resuspended in freshly prepared Seahorse assay medium, diluted in the assay medium to working concentrations (1 µmol/l oligomycin, 0.8 µmol/l FCCP and 0.5 µmol/l rotenone/antimycin A) and injected into respective ports (port A, oligomycin; port B, FCCP; port C, rotenone/antimycin A; and port D, empty). Medium in the plate was changed to Seahorse assay medium and the cells were incubated for 1 h in a non-CO_2_ incubator. The Seahorse assay was then run according to pre-set cycles designed on the Wave app (5 measurement cycles for all compounds) and analysed.

### Immunofluorescence and morphometry on tissue sections and cells

Human pancreas, as well as mouse and human islets embedded in Optimal Cutting Temperature compound (OCT) medium, were sectioned at 7 µm thickness, fixed in 4% (vol./vol.) paraformaldehyde (PFA), washed with PBS and permeabilised with 0.2% Triton (Sigma-Aldrich). Sections were then blocked in 2% BSA and incubated overnight at 4°C with the relevant primary antibodies (listed in ESM Table 2). Sections were then probed with respective fluorophore-tagged secondary antibodies (ESM Table 3) for 1 h at room temperature, stained with DAPI (1 µg/ml; Sigma-Aldrich), mounted (Dako, Agilent, Denmark) and cover-slipped.

MIN6 cells were seeded on attachment-factor-coated coverslips in a 12-well plate. Cells were washed, fixed with 4% PFA, permeabilised with 0.5% Triton and blocked in 2% BSA prior to incubating with primary antibodies overnight at 4°C (ESM Table 2). Cells were then incubated with fluorophore-tagged secondary antibodies for 1 h at room temperature, stained with DAPI and mounted onto microscope slides. Imaging was performed on an Olympus VS120 or Leica SP5 Confocal microscope (Olympus Life Science Solutions, Japan) and intensity or positive cell numbers were measured using ImageJ. Measurement of corrected total cell fluorescence is outlined in ESM Methods.

### Western blotting

Cells were homogenised in cold RIPA buffer (Cell Signalling Technology) containing protease inhibitor cocktail (Sigma-Aldrich) and phosphatase inhibitor cocktail (Roche Applied Science, Hercules, CA, USA). Protein was quantified using a DC assay (BioRad, Hercules, CA, USA), denatured using Laemmli SDS buffer heated to 95°C for 5 min, and subjected to SDS-PAGE and then transferred onto PVDF membrane (Millenium Science, VIC, Australia) as described previously [19]. Membranes were probed (antibodies are listed in ESM Table 2), visualised on an Odyssey CLx Imaging System (Licor, Lincoln, NE, USA) and band intensity evaluated using ImageJ normalised to the housekeeping gene.

### RNA extraction and quantification by reverse-transcription quantitative PCR

For reverse-transcription quantitative PCR (RT-qPCR), RNA was extracted using ISOLATE-II RNA MiniKits (Bioline, London, UK) with on-column DNase treatment. RNA was quantified using a Nanodrop (BioTek, Winooski, VT, USA) and reverse-transcribed using a SensiFAST cDNA synthesis kit (Bioline). cDNA was amplified in triplicate with commercially available gene-specific hydrolysis probes (Taqman primers, listed in ESM Table 4, Invitrogen, Carlsbad, CA, USA) using a CFX384 PCR machine (BioRad). Thermal cycling conditions were 95°C for 2 min, followed by 40 cycles of 95°C for 10 s and 60°C for 30 s. Data were analysed using the ${2}^{{-\Delta \Delta \text{C}}_{\text{t}}}$ method, with expression normalised to the reference gene (18S) and also to the referent control group.

### Single-cell RNA-seq analysis

Single-cell RNA-seq data were accessed from the Human Pancreas Analysis Program (HPAP-RRID:SCR_016202) Database (https://hpap.pmacs.upenn.edu/). Detailed methodology is outlined in ESM Methods.

### Statistical analysis

Data are presented as the mean ± SD from at least three independent cell cultures, comprising *n*=3 or 4 animals or *n*=3–6 human samples per group. Differences between the two groups were assessed using two-tailed Student’s *t* tests. When comparing more than two groups, significance was calculated using one-way or two-way ANOVA, depending upon the number of independent variables, followed by Tukey’s post hoc testing for multiple comparisons, using GraphPad Prism software v.10 (San Diego, CA, USA). *p*<0.05 was considered statistically significant.

## Results

### CD47 and TSP1 reflect islet response to hypoxia

**Fig. 1:**
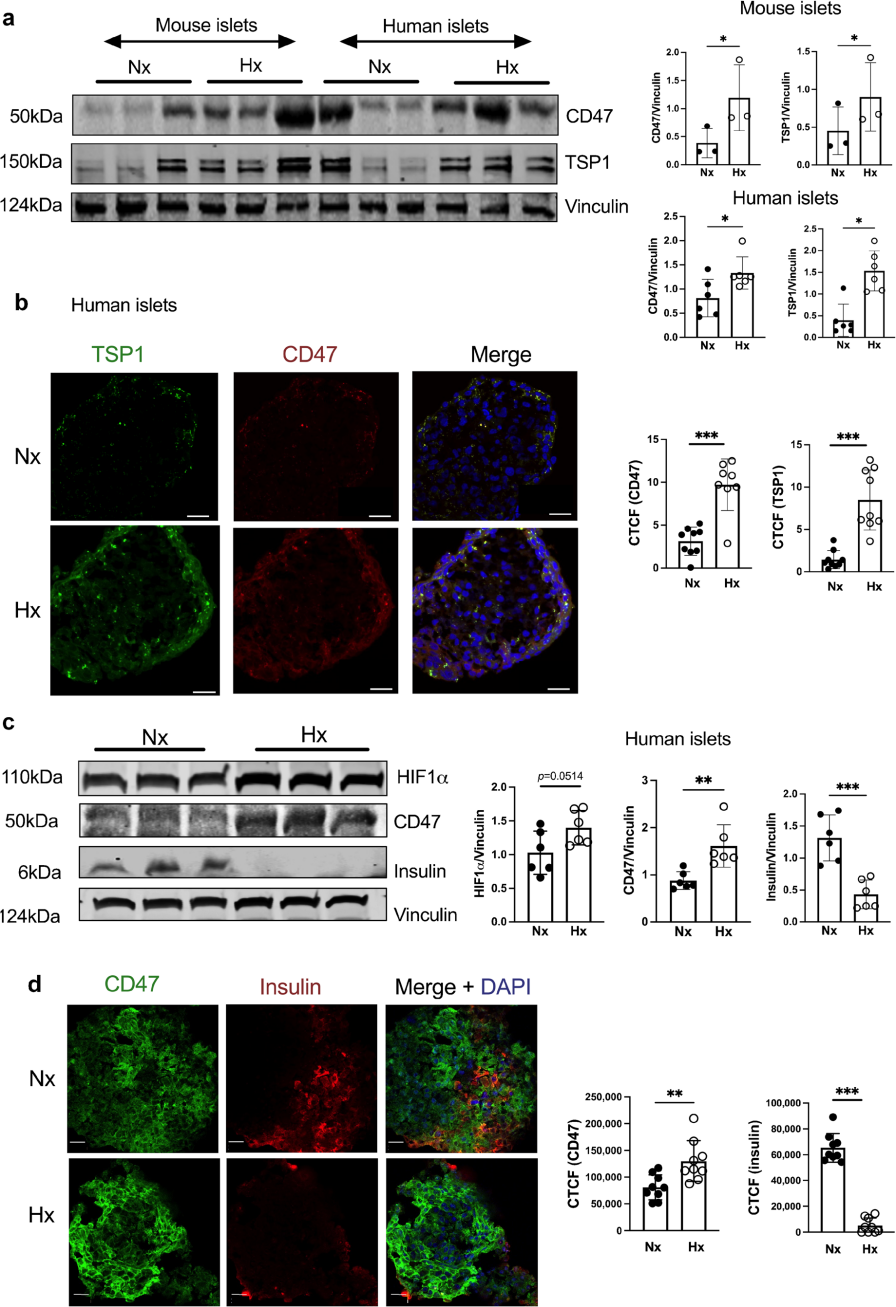
Hypoxia upregulates TSP1–CD47 signalling and limits insulin production in islets. (**a**) Mouse (*n*=3 replicates from *n*=3 or 4 mice per replicate) and human islets (*n*=6 donors) were subjected to normoxia (F_I_O_2_ 21%) or hypoxia (F_I_O_2_ 1%) for 24 h. Lysates were collected, resolved by SDS-PAGE and probed for CD47 and TSP1. Representative western blot and combined densitometry are shown. (**b**) Isolated human islets subjected to normoxia or hypoxia were embedded in OCT, sectioned and stained for CD47 (red), TSP1 (green) and DAPI (blue). Scale bar, 20 μm; magnification ×60. Quantification of staining from *n*=3 donors from two–three randomly chosen regions-of-interest per image. (**c**) Human islets (*n*=6 donors) subjected to normoxia or hypoxia were resolved by SDS-PAGE and probed for HIF1α, CD47 and insulin. Representative western blot and combined densitometry are shown. (**d**) Human islets (*n*=3 donors) subjected to normoxia or hypoxia were embedded in OCT, sectioned and stained for CD47 (green), insulin (red) and DAPI (blue). Quantification of staining from three randomly chosen regions-of-interest per image. Scale bar, 20 μm; magnification ×60. All data are mean ± SD. *p<0.05, **p<0.01 and ***p<0.001 by Student’s *t* test. CTCF, corrected total cell fluorescence; Hx, hypoxia; Nx, normoxia

Hypoxia limits islet viability and function during isolation and purification, as well as following heterotopic transplantation into the liver [5]. Beta cell hypoxia is also evident in vivo in preclinical models of type 2 diabetes [20] and this effect can be replicated by hyperglycaemic conditions in vitro [21]. TSP1 has known hypoxia response elements in the proximal promoter region [13]. Primary murine islets from C57BL/6 mice and human islets exposed to hypoxia for 24 h demonstrated increased CD47 and TSP1 protein expression (Fig. 1a), and this was replicated with immunofluorescent staining in isolated human islets (Fig. 1b). In subsequent experiments, human islets exposed to hypoxia displayed increased hypoxia inducible factor (HIF) 1α and CD47 expression, with concurrent suppression of insulin (Fig. 1c), confirmed by immunofluorescent staining (Fig. 1d).

### Hypoxic impairment of beta cell insulin secretion can be improved by targeting CD47

**Fig. 2:**
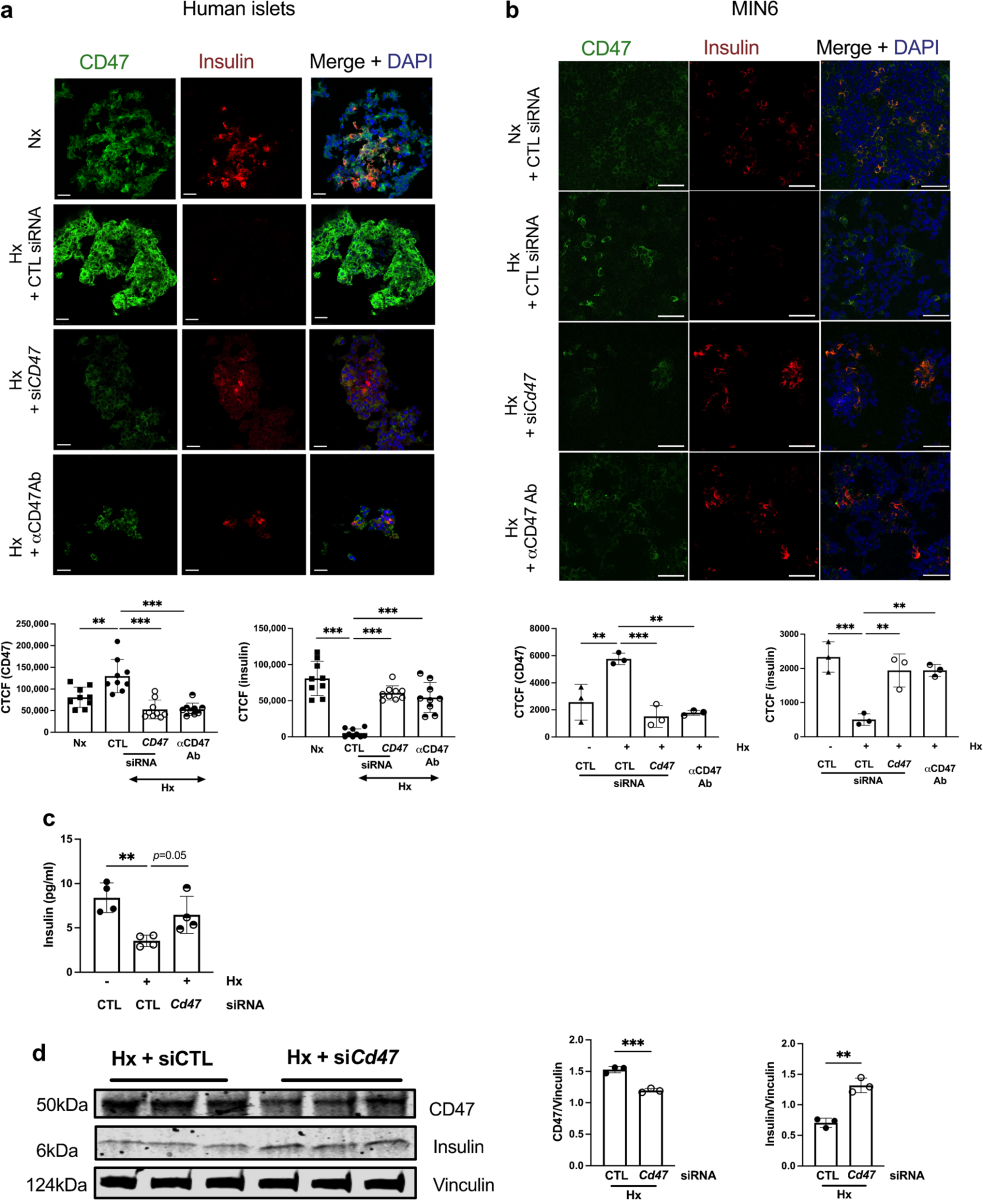
Hypoxic upregulation of CD47 impairs insulin secretion from islets. (**a**) Human islets transfected with non-silencing control or *CD47* siRNA, or treated with anti-CD47 antibody were subjected to normoxia or hypoxia (F_I_O_2_ 1%) for 24 h and stained for CD47 (green), insulin (red) and DAPI (blue). Images were obtained on a confocal microscope at ×60 magnification. Scale bar, 20 µm. Corrected total cell fluorescence was quantified from *n*=3 donors from three randomly selected regions-of-interest per image. (**b**) MIN6 cells transfected with non-silencing control or *Cd47* siRNA, or pre-treated with anti-CD47 antibody were subjected to normoxia or hypoxia for 24 h and stained for CD47 (green), insulin (red) and DAPI (blue). Images were obtained on a confocal microscope at ×60 magnification. Scale bar, 50 µm. Corrected total cell fluorescence was quantified from *n*=5 randomly chosen regions-of-interest from *n*=3 replicates. (**c**) Insulin concentration in supernatant fractions from MIN6 cell culture (*n*=4 replicates) assessed by ELISA. (**d**) MIN6 cells transfected with non-silencing control or *Cd47* siRNA were exposed to hypoxia (F_I_O_2_ 1%) for 24 h. Lysates were collected, resolved by SDS-PAGE and probed for CD47 and insulin. Representative western blot and combined densitometry are shown. All data are mean ± SD. **p<0.01 and ***p<0.001 by Student’s *t* test (**d**), or one-way ANOVA (**a**, **b**, **c**). α, anti-; Ab, antibody; CTCF, corrected total cell fluorescence; CTL, control; Hx, hypoxia; Nx, normoxia

We have previously shown that genetic lack of CD47 signalling can be replicated pharmacologically to preserve insulin secretion [16]. We investigated whether this effect could be leveraged to optimise insulin secretagogue activity in hypoxic conditions that compromise glucose control. Human islets exposed to hypoxia upregulated CD47 with concurrent suppression of insulin (when compared with normoxic controls), and pre-treatment with siRNA to *CD47* or antibody targeting CD47 increased insulin expression (Fig. 2a). This effect was replicated in MIN6 cells following immunofluorescence staining (Fig. 2b) and measurement of insulin secretion by ELISA (Fig. 2c), and was confirmed by western blot (Fig. 2d).

### CD47 downregulation prevents hypoxia-induced beta cell apoptosis and senescence

**Fig. 3:**
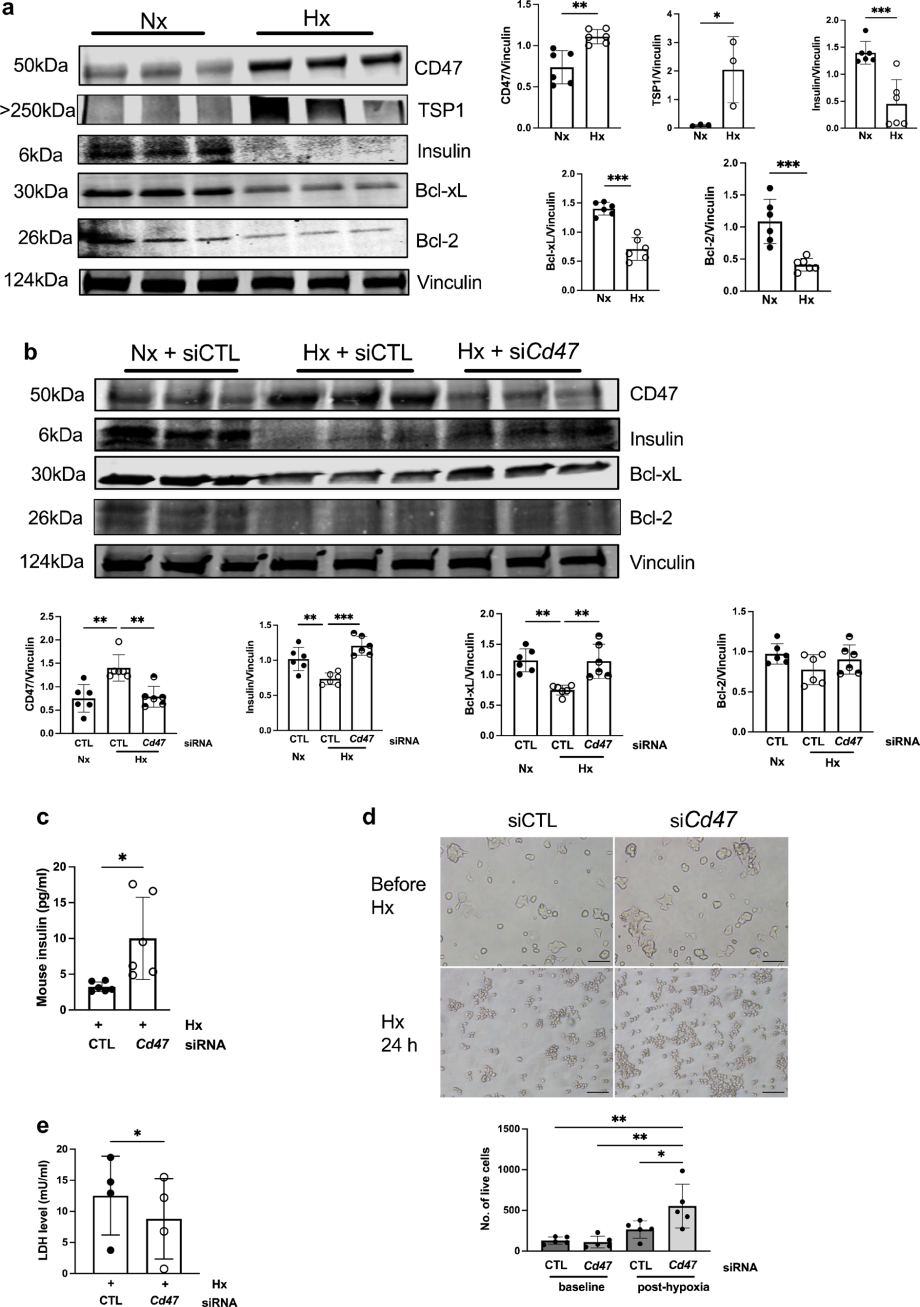
Hypoxia promotes apoptosis in islets that is mitigated by limiting CD47 signalling. (**a**) MIN6 cells (*n*=3–6 replicates) exposed to normoxia (F_I_O_2_ 21%) or hypoxia (F_I_O_2_ 1%) for 24 h were resolved by SDS-PAGE and probed for CD47, TSP1, insulin, Bcl-2 and Bcl-xL. Representative western blot and combined densitometry are shown. (**b**) MIN6 cells transfected with non-silencing control or *Cd47* siRNA were exposed to normoxia (Nx, F_I_O_2_ 21%) or hypoxia (Hx, F_I_O_2_ 1%) for 24 h. Lysates (*n*=6 replicates) were collected, resolved by SDS-PAGE and probed for CD47, insulin, Bcl-2 and Bcl-xL. (**c**) Insulin concentrations in supernatant fractions from cell cultures were assessed by ELISA (*n*=6 replicates). (**d**) Cell numbers before and after 24 h hypoxia (*n*=5 replicates). Images were obtained on a brightfield microscope at ×4 magnification. Scale bar, 200 µm. (**e**) LDH levels in hypoxic cell culture after 24 h (*n*=4 replicates). All data are mean ± SD. *p<0.05, **p<0.01 and ***p<0.001 by Student’s *t* test (**a**, **c**, **e**), one-way ANOVA (**b**) or two-way ANOVA (**d**). CTL, control; Hx, hypoxia; Nx, normoxia

Cell survival is tightly regulated by signalling pathways that converge on anti-apoptotic proteins from the B cell lymphoma (Bcl) family. Culturing islets compromises viability by inducing apoptosis, and pro-survival proteins Bcl-2 and Bcl-xL are implicated in islet survival under stress [22]. MIN6 cells cultured under hypoxic conditions demonstrated suppression of anti-apoptotic factors Bcl-xL and Bcl-2 (Fig. 3a). Knockdown of CD47 using siRNA restored Bcl-xL and Bcl-2 expression (Fig. 3b) and increased insulin secretion into the supernatant fraction (Fig. 3c) when compared with cells cultured with control siRNA. Proliferation under hypoxic conditions was also increased in MIN6 cells pre-treated with *Cd47* siRNA (Fig. 3d) and LDH secretion into culture supernatant was significantly reduced (Fig. 3e).

**Fig. 4:**
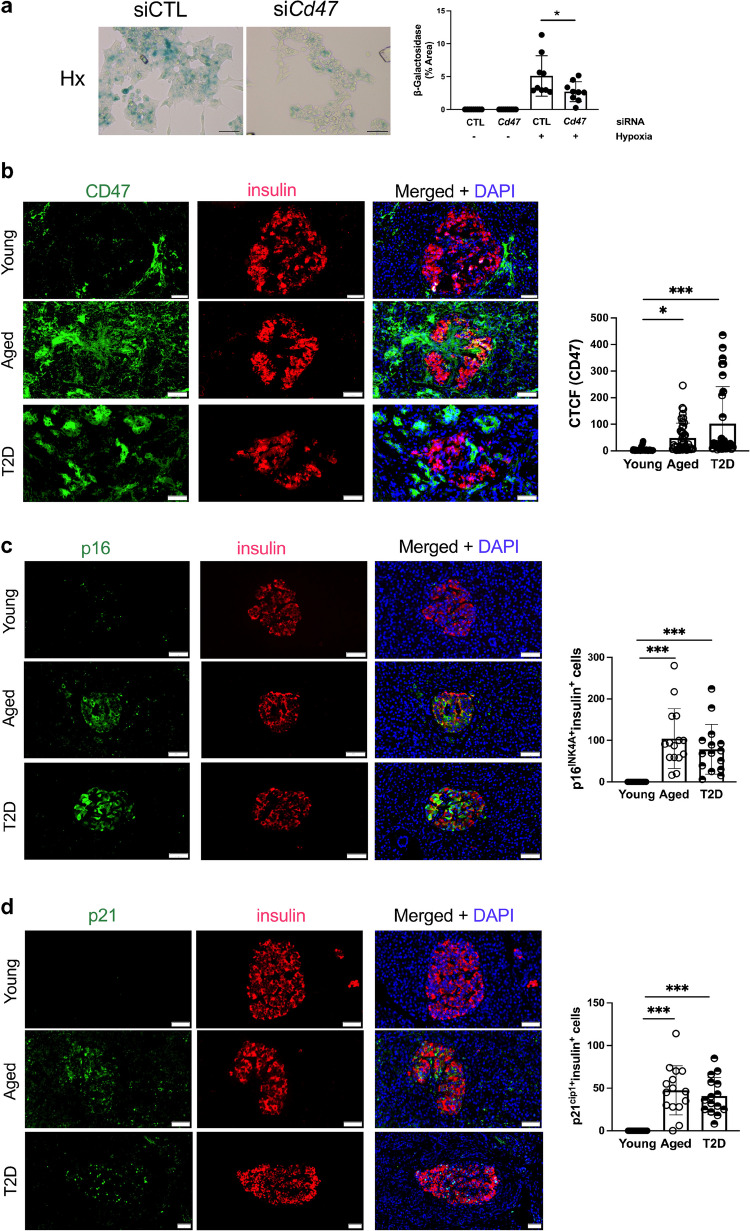
Senescence and CD47 are upregulated by ageing and diabetes. (**a**) MIN6 cells were cultured in six-well plates, transfected with non-silencing control or *Cd47* siRNA and then exposed to normoxia (F_I_O_2_ 21%) or hypoxia (F_I_O_2_ 1%) for 24 h. Senescence-associated β-galactosidase activity was measured from three regions-of-interest per well from *n*=3 replicates. Images were obtained on a brightfield microscope at ×20 magnification. Scale bar, 50 µm. (**b**–**d**) Human pancreas samples from non-diabetic young (<35 years) and aged (>60 years) organ donors, as well as donors with type 2 diabetes were stained for insulin and CD47 (**b**), insulin and p16^INK4A+^ (**c**) or insulin and p21^cip1+^ (**d**). Corrected total cell fluorescence for CD47 was quantified from *n*=5 islets per sample (*n*=3 donors per group). p16^INK4A+^ or p21^cip1+^ cells within the islets of Langerhans (*n*=5 islets per sample) were counted (**c**, **d**). Images were obtained on a confocal microscope at ×60 magnification. Scale bar, 50 µm. All data are mean ± SD. *p<0.05 and ***p<0.001 by two-way ANOVA (**a**) or one-way ANOVA (**b**, **c**, **d**). CTCF, corrected total cell fluorescence; CTL, control; Hx, hypoxia; Nx, normoxia; T2D, type 2 diabetes

We have reported previously that ageing is associated with increased TSP1–CD47 signalling [23] and more recently showed that TSP1 induces a senescence-associated secretory phenotype [24] that appears to be CD47-dependent [25]. Hypoxia increased senescence-associated β-galactosidase staining in MIN6 cells; this increase was mitigated by pre-treatment with siRNA to *CD47* (Fig. 4a). We next investigated whether we could detect changes in senescence and CD47 expression in human endocrine pancreas. CD47 expression was increased in the endocrine pancreas in aged and diabetic individuals (Fig. 4b). The number of p16^INK4A+^ and p21^cip1+^ (cyclin-dependent kinase inhibitors 2A and 1A, respectively) beta cells (with colocalised insulin staining), indicative of senescence, was significantly increased in both older donors and donors with type 2 diabetes (Fig. 4c, d and ESM Fig. 1).

### CD47 impairs autophagy and self-renewal capacity in beta cells

**Fig. 5:**
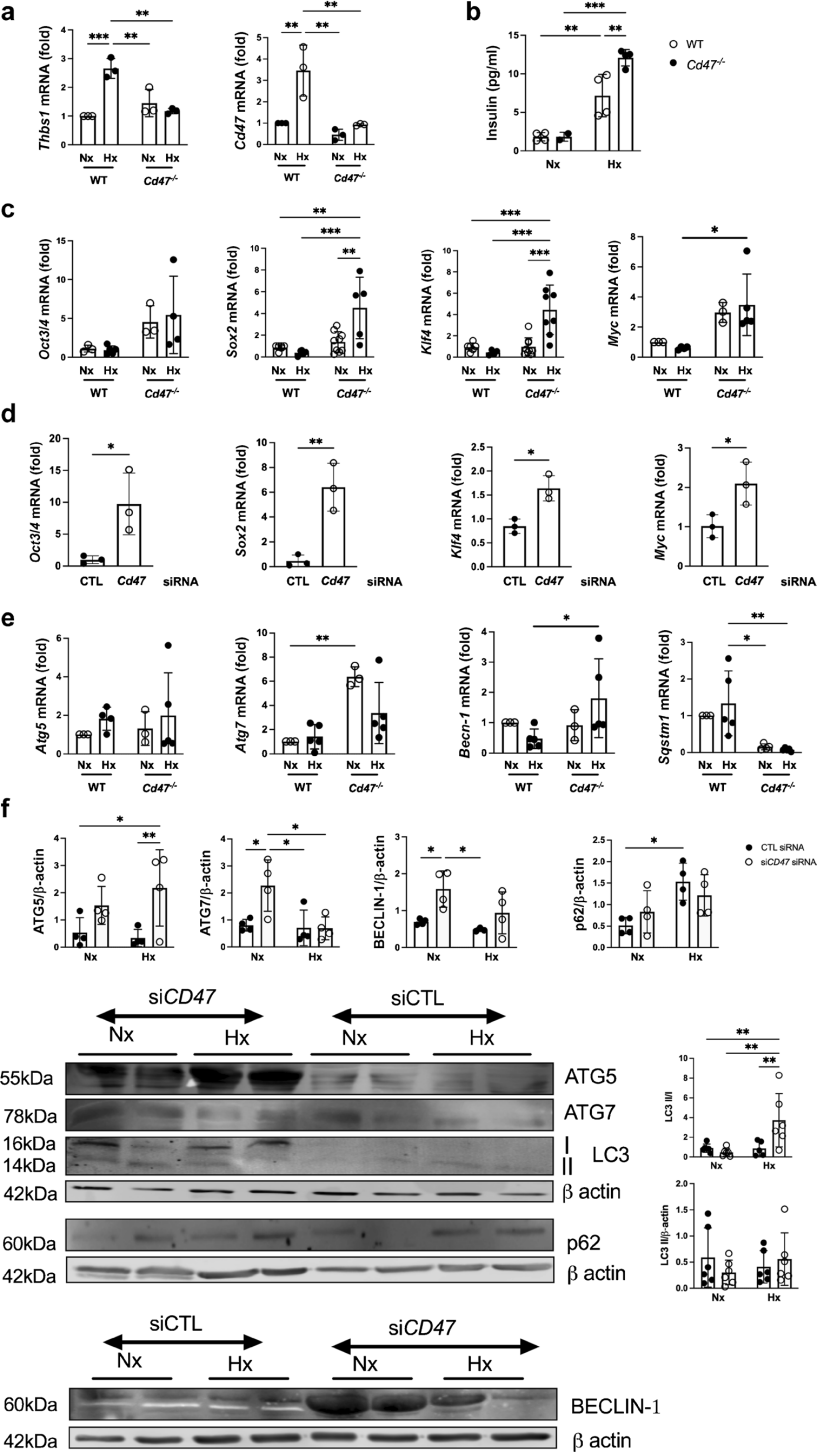
CD47 signalling limits autophagy and self-renewal expression in islets. WT and *Cd47*^−/−^ mouse islets (from *n*=3–5 samples isolated from *n*=3 or 4 mice/sample) were isolated and subjected to normoxia (F_I_O_2_ 21%) or hypoxia (F_I_O_2_ 1%) for 24 h. (**a**) mRNA expression of *Thbs1* and *Cd47* was measured by RT-qPCR. (**b**) Insulin concentration in the supernatant fraction was measured by ELISA. (**c**, **d**) RT-qPCR for self-renewal factors *Oct3/4*, *Sox2*, *Klf4* and *Myc* (OSKM) in primary mouse islets following normoxia or hypoxia for 24 h (**c**) or in MIN6 cells under normoxia transfected with control or *Cd47* siRNA (**d**) was performed. Transcripts were normalised to 18S and then to the referent control (WT normoxic cells or control value respectively). (**e**) RT-qPCR analysis of autophagy markers *Atg5*, *Atg7*, *Becn1* (*Beclin-1*) and *Sqstm1* (*p62*) in primary murine islet subjected to normoxia or hypoxia. (**f**) Human islets transfected with non-silencing control or *Cd47* siRNA then exposed to normoxia or hypoxia for 24 h, protein resolved by SDS-PAGE and probed for ATG5, ATG7, BECLIN-1, LC3 and p62. Representative western blot and combined densitometry are shown. All data are mean ± SD. *p<0.05, **p<0.01 and ***p<0.001 by two-way ANOVA (**a**, **b**, **c**, **e**, **f**) or Student’s *t* test (**d**). CTL, control; Hx, hypoxia; LC3, microtubule-associated protein light chain 3; Nx, normoxia

Previous work has demonstrated that *Cd47*^−/−^ mice display limited cellular damage in response to injury [26, 27], as do primary endothelial cells, epithelial cells and vascular smooth muscle cells, via several molecular pathways that provide cytoprotection [28]. Assessment of primary murine islets subjected to normoxia or hypoxia demonstrated upregulation of *Thbs1* and *Cd47* mRNA restricted to WT cells under hypoxic conditions (Fig. 5a), and increased insulin was detected in the supernatant fraction from hypoxic *Cd47*^−/−^ mouse islets (Fig. 5b). Viability (only assessed under normoxic conditions) was improved in the absence of CD47 expression (ESM Fig. 2). Human islets are terminally differentiated and do not replicate; however, replication is a feature of islet regeneration [29] in adult mice. Self-renewal capacity, as well as pluripotency, is driven by organic cation/carnitine transporter 4 (encoded for by *Oct3/4*, also known as *Pou5f1*), SRY-box transcription factor 2 (*Sox2*), Kruppel-like factor 4 (*Klf4*) and myelocytomatosis oncogene (*Myc*), known collectively as OSKM. *Cd47*^−/−^ mice have clear evidence of upregulated OSKM transcription factors without increased stem-cell populations [11], and this phenomenon was replicated in *Cd47*^−/−^ mouse islets under both normoxic and hypoxic conditions (Fig. 5c). These findings were recapitulated in MIN6 cells under standard culture conditions in cells transfected with siRNA to *Cd47* (Fig. 5d).

Autophagy (the lysosomal degradation of unwanted cellular components) is indispensable for islet homeostasis and survival, enabling resistance to ER stress and DNA damage. Autophagy-deficient beta cells display increased susceptibility to diabetes [30, 31]. mRNA expression of *Atg7* was upregulated in *Cd47*^−/−^ mouse islets, and this persisted under hypoxia (Fig. 5e). *Becn1* (Beclin-1) transcript demonstrated hypoxic upregulation, with no change in *Atg5*. *Sqstm1*, which encodes p62, a protein that accumulates when autophagy is limited, was significantly downregulated in Cd47^−/−^ islets. Assessment of the autophagic profile in human islets revealed increased autophagy-related 7 (ATG7) and beclin-1 protein expression under normoxia, and upregulated autophagy-related 5 (ATG5) and microtubule-associated protein light chain 3 (LC3) II/I ratios under hypoxic stimulus following treatment with *Cd47* siRNA (Fig. 5f). These data confirm some cytoprotective mechanisms are driven by enhanced autophagy with genetic absence of CD47.

### Limiting CD47 signalling protects against UPR activation in beta cells

**Fig. 6:**
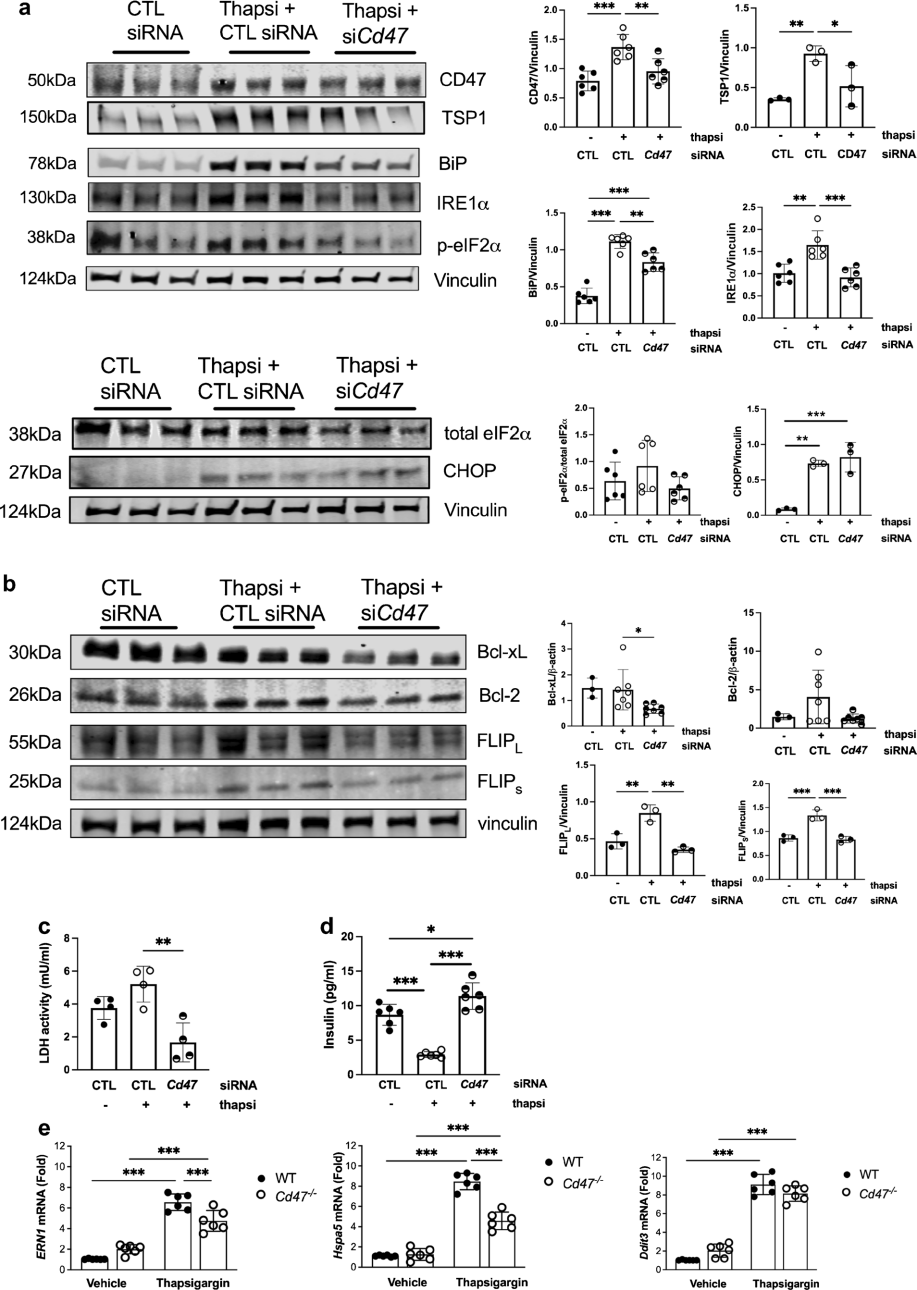
Loss of CD47 signalling limits ER stress in islets. (**a**, **b**) MIN6 cells transfected with non-silencing control or *Cd47* siRNA were exposed to thapsigargin for 18 h. Cell lysates were resolved by SDS-PAGE and probed for TSP1, CD47, BiP, IRE1α, total and phospho-eIF2α, and CHOP (**a**), or Bcl-xL, Bcl-2 and FLIP (long [FLIP_L_] and short [FLIP_S_] isoforms) (**b**). Representative western blot and combined densitometry from *n*=3–6 replicates are shown. (**c**, **d**) LDH activity (**c**) and insulin content (**d**) in cell culture supernatant fractions were measured by ELISA. (**e**) Primary murine islets from WT and *Cd47*^−/−^ mice were isolated and exposed to thapsigargin for 18 h. mRNA expression of *ERN1* (*Ire1α*), *Hspa5* (*BiP*), and *Ddit3* (*CHOP*) was measured by RT-qPCR, normalised by the mean of 18S, and then vehicle-treated WT cells were used as the referent control. All data are mean ± SD. *p<0.05, **p<0.01 and ***p<0.001 by one-way ANOVA (**a**–**d**) or two-way ANOVA (**e**). CTL, control; Hx, hypoxia; Nx, normoxia; Thapsi, thapsigargin

Induction of ER stress leads to activation of the UPR to restore cellular homeostasis. The first cellular events in the UPR cascade include PERK-mediated phosphorylation of the translation initiation factor eukaryotic initiation factor 2α (eIF2α), leading to the reduction of global protein synthesis, and activation of the transmembrane protein inositol-requiring enzyme 1 α (IRE1α) [32]. These pathways upregulate transcription of UPR target genes, such as that encoding the ER chaperone binding immunoglobulin protein (BiP), in an attempt to restore protein folding homeostasis [33]. As we had observed protection of islet function and viability under hypoxic conditions by limiting CD47 signalling, we investigated whether the cytoprotective effect was applicable to other exogenous stressors. MIN6 cells exposed to thapsigargin, a non-competitive inhibitor of the sarco/ER Ca^2+^-ATPase, showed upregulated expression of CD47 and TSP1, as well as phosphorylated-eIF2α, IRE1α, BiP and C/EBP homologous protein (CHOP) (Fig. 6a). Expression of all factors, except CHOP, was significantly decreased by CD47 knockdown. The anti-apoptotic markers Bcl-2 and FLICE-inhibitory protein (FLIP) short and long isoforms were also increased in response to thapsigargin and the increase was mitigated by *Cd47* siRNA (Fig. 6b). Bcl-xL expression was not significantly different after thapsigargin exposure. These findings correlated with levels of LDH in cell culture supernatant fractions (Fig. 6c) and insulin secretory activity under the same conditions (Fig. 6d). *Cd47*^−/−^ compared with WT mouse islets, demonstrated decreased *Ire1α* (also known as *Ern1*) and *BiP* (also known as *Hspa5*) mRNA in response to thapsigargin (Fig. 6e). *Chop* (also known as *Ddti3*) mRNA levels were unchanged.

### TSP1 and CD47 signalling in diabetes

**Fig. 7:**
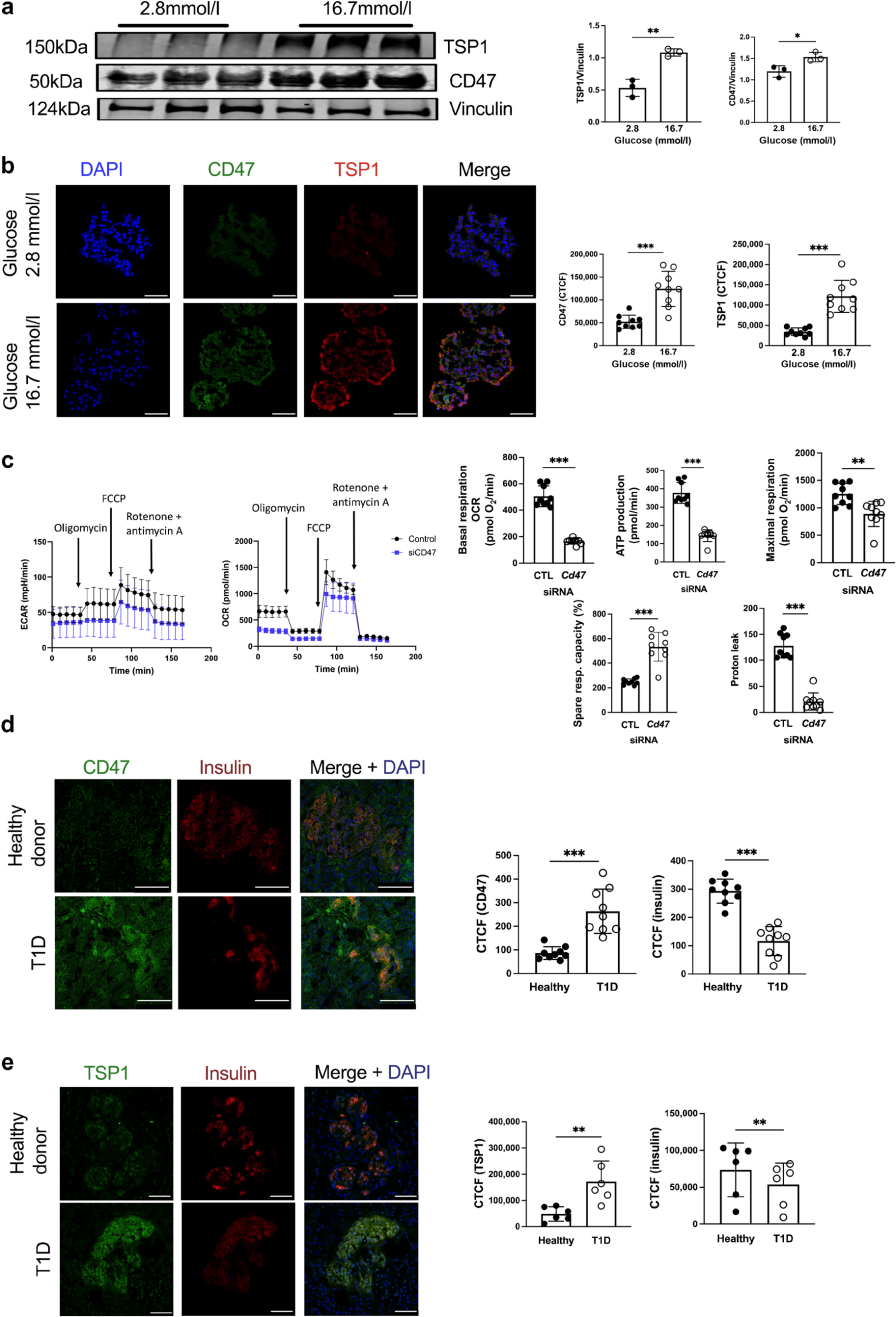
TSP1–CD47 signalling is upregulated in type 1 diabetes. Human islets were cultured at baseline glucose (2.8 mmol/l) or hyperglycaemic (16.7 mmol/l) conditions. (**a**) Cell lysates were resolved by SDS-PAGE and probed for TSP1 and CD47. Representative western blot and combined densitometry are shown from *n*=3 replicates. (**b**) Islets were embedded in OCT, sectioned and stained for CD47 (green), TSP1 (red) and DAPI (blue). Scale bar, 50 μm; magnification ×60. Quantification of staining from *n*=3 donors from three randomly chosen regions-of-interest per image. (**c**) MIN6 cells transfected with non-silencing control or *Cd47* siRNA were used to seed a Seahorse assay plate (10^5^ cells/well). The ECAR and OCR was measured using the Seahorse XFe24 Well Analyzer in hyperglycaemic media (16.7 mmol/l), with the addition of oligomycin (1 µmol/l), FCCP (0.8 µmol/l) and rotenone/antimycinA (0.5 µmol/l) at time points as indicated. Basal respiration, ATP production, maximal respiration, spare respiratory capacity and proton leak from *n*=3 replicates run in triplicate were calculated. Healthy donor pancreas (*n*=3) or pancreas from a donor with type 1 diabetes (*n*=1) was stained for (**d**) CD47 and insulin or (**e**) TSP1 and insulin. Scale bar, 75 μm; magnification ×60. Corrected total cell fluorescence was quantified averaged from *n*=9 or *n*=6 islets of Langerhans (respectively) in the endocrine pancreas. *p<0.05, **p<0.01 and ***p<0.001 by Student’s *t* test. CTCF, corrected total cell fluorescence; CTL, control; T1D, type 1 diabetes

TSP1 is upregulated in endothelial cells in response to hyperglycaemia [15] and we replicated this finding in human islets, demonstrating increased TSP1 and CD47 expression by western blot (Fig. 7a) and immunofluorescent staining (Fig. 7b). Mitochondrial respiration and insulin secretion are altered under pathological conditions where beta cell mass is limited, including diabetes and islet transplantation. Since islets couple glucose metabolism to insulin release through the mitochondrial electron transport and ATP production, we assessed extracellular acidification rate (ECAR) and oxygen consumption rate (OCR), and the effect of CD47 expression on cellular respiration in MIN6 cells. Basal respiration, ATP production, maximal respiration and proton leak were all significantly reduced in *Cd47* siRNA-treated cells, although spare respiratory capacity was elevated (Fig. 7c), suggesting superior mitochondrial integrity. We previously demonstrated upregulation of CD47 expression in endocrine pancreas of NOD mice [16] and now showed that both CD47 and TSP1 (Fig. 7d) were upregulated in human pancreas from donors with type 1 diabetes compared with healthy non-diabetic donors.

**Fig. 8:**
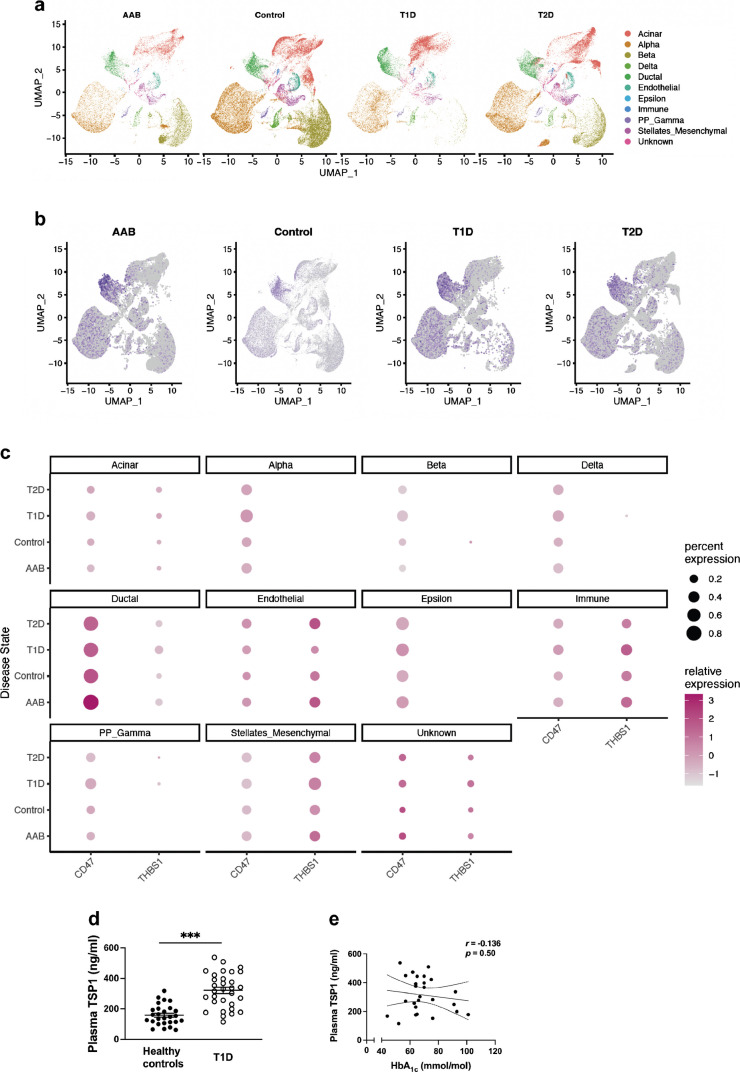
TSP1–CD47 signalling is upregulated in type 1 and type 2 diabetes. (**a**) UMAP of pancreatic cell populations across disease states, including autoantibody-positive, healthy (control), type 1 diabetes and type 2 diabetes. Each dot represents an individual cell, coloured by cell type. (**b**) UMAP of CD47 expression in cells from autoantibody-positive donors, healthy control donors and donors with type 1 diabetes and type 2 diabetes. Grey dots represent cells with low or undetectable CD47 expression; purple dots indicate cells with higher expression levels. (**c**) Dot plot showing the relative expression of *CD47* and *THBS1* across different cell types and disease states. The size of the dots indicates the percentage of cells expressing each marker, while the colour intensity represents the average expression level. (**d**) Plasma TSP1 levels from healthy volunteers (*n*=27) or individuals with type 1 diabetes (*n*=32) and normal renal function, measured by ELISA. (**e**) Linear regression analysis of plasma TSP1 levels and HbA_1c_ in individuals with type 1 diabetes. All data are mean ± SD. ***p<0.001 by Student’s *t* test. AAB, autoantibody-positive; PP, pancreatic polypeptide; T1D, type 1 diabetes; T2D, type 2 diabetes; UMAP, uniform manifold approximation and projection

CD47 demonstrates universal cellular expression and TSP1 is upregulated in all nucleated cells. However, immunofluorescence staining is limited in its capacity to accurately distinguish and compare expression in all endocrine cells within the islets. Therefore, we analysed single-cell RNA-seq data from the Data Portal of the Human Pancreas Analysis Program (PANC-DB, https://hpap.pmacs.upenn.edu/) to determine relative transcript expression in pancreas from healthy control individuals, autoantibody-positive (non-diabetic) individuals and individuals with type 1 diabetes and type 2 diabetes. Uniform manifold approximation and projection (UMAP) of pancreatic cell populations (Fig. 8a) and CD47 expression (Fig. 8b) were derived, demonstrating clear upregulation in alpha, beta and delta endocrine cells in autoantibody-positive and diabetes samples. These findings were confirmed using a dot plot displaying relative expression of *CD47* and *THBS1* across cell types and disease states (Fig. 8c). CD47 expression was increased in all endocrine cells in diabetes, with the highest expression in ductal cells (this did not appreciably change with disease state). Upregulation of *TSP1* was seen in type 2 diabetes endothelial cells, with contributions from stellate/mesenchymal and immune cell populations.

Finally, we considered whether TSP1 could be a potential biomarker of hyperglycaemia, measuring platelet-poor plasma TSP1 levels in healthy volunteers with no known medical conditions (‘healthy controls’) and individuals with type 1 diabetes without evidence of renal microvascular complications, including normal eGFR (>90 ml/min per 1.73m^2^) and urine albumin/creatinine ratio (<2.5 mg/mmol). The age of participants was not significantly different (31.2 ± 14.4 years for healthy individuals vs 34.5 ± 17.5 years for individuals with type 1 diabetes; χ^2^ test: 2, *n*=59, *p*=0.611) but healthy controls were more likely to be self-reported female (χ^2^ test: 2, *n*=59, *p*<0.01). Plasma TSP1 was significantly higher in type 1 diabetes (Fig. 8d), but did not correlate with HbA_1c_ (Fig. 8e).

## Discussion

Here we demonstrate a multifaceted role for CD47 in protecting islet viability and function, which remains consistent in response to different exogenous stressors. Using genetic and pharmacological approaches to modulate CD47 signalling capability, we reveal that CD47 inhibition improves anti-apoptotic, senescence, autophagic and self-renewal responses to hypoxia, hyperglycaemia or ER stress in MIN6 cells, as well as primary mouse and human islets. We also show that TSP1 is likely the soluble ligand involved in CD47 activation, with clear upregulation in islets under these conditions, as well as in the human pancreas affected by type 1 diabetes. These findings complement our previous work demonstrating that CD47 also tonically limits insulin secretion [16], and emphasise that CD47 potentially represents a novel target to preserve or maximise beta cell function, particularly in circumstances of limited islet mass.

The high metabolic activity of islets dictates high oxygen consumption to maintain function and consequently they are exquisitely sensitive to hypoxia. Islet viability and responsiveness to hyperglycaemia are strongly linked to oxygen tension [34]. Changes in transcriptional profiles in hypoxic islets are coordinated by HIF, and studies demonstrate that HIF1α activation impairs beta cell function (and consequent glucose control) by switching glucose metabolism from aerobic oxidative phosphorylation to anaerobic glycolysis [35, 36]. We demonstrate concomitant upregulation of HIF1α, CD47 and TSP1 in response to hypoxia, with concomitant inhibition of insulin secretion, an effect that can be reversed by RNA interference or antibody against CD47. HIF has also been shown to activate transcription of CD47 in hypoxic malignant cells [37], although this was only in the context of enhanced phagocytosis related to the ‘don’t-eat-me’ signal. It is possible that HIF independently upregulates CD47 expression to further limit insulin secretion under hypoxic conditions and this requires further investigation.

Oxygen deprivation is consistently observed in pancreatic islets upon isolation and transplantation. Separation of islets from the exocrine tissue and the extracellular matrix leads to disruption of the vascular access, leaving diffusion as the only mechanism that provides oxygen; this is insufficient for highly metabolically active cells. This effect persists until neo-angiogenesis has developed. Pre-existing intra-islet hypoxia is associated with unfavourable transplantation outcomes. Primary islets and beta cell lines readily become hypoxic under high-glucose conditions [38], and this is recapitulated in vivo in animal models of type 2 diabetes, and driven by HIF1α [39]. Previous work has shown that islet OCR increases significantly as glucose concentrations rise [40], correlating with metabolic activity and delineating differences in functional viability. Downregulation of CD47 in MIN6 cells cultured under high-glucose concentrations demonstrated improved spare respiratory capacity and decreased proton leak, suggesting improved metabolic activity and functional viability, particularly mitochondrial integrity.

The UPR is a highly conserved cellular response pathway in the ER, aimed at preservation of correct protein folding and protein load, governing the balance between cellular survival and death. These processes are integral to beta cell physiology [41]. Chronic UPR activation resulting from ER stress that cannot be mitigated is maladaptive, promoting cell cycle arrest and senescence that culminates in apoptosis, protecting additional parenchymal cells from exposure to damaging secretory proteins. Under conditions of elevated insulin demand, coupled with hypoxia and hyperglycaemia within the endocrine pancreas, the homeostatic UPR response is exhausted and beta cells become complicit in their own demise. Modulating CD47 expression has been shown to limit cellular dysfunction from reactive oxygen species, as well as Fas-mediated cell death through changes in mitochondrial membrane potential. As expected, genetic deletion or pharmacological modification of CD47 expression in beta cells reduced the UPR and apoptotic responses to thapsigargin. Hypoxia also promotes ER stress and activates the UPR to control signalling pathways including autophagy and self-renewal. Our data are consistent with a cytoprotective effect related to downregulation of CD47 activity (likely through TSP1) that preserves cellular functions, reduces UPR activation, prevents cell death and maintains insulin secretion. The chaperone glucose-regulated protein 78 (GRP78/BiP) is a master regulator of UPR and has also been shown to modify CD47 expression and tumour growth [42], suggesting a potential bidirectional role. The numerous effects of CD47 blockade on cell survival and preservation of function is not completely understood and differential regulation of autophagy, self-renewal and senescence in beta cells may occur through crosstalk with the UPR pathway.

In diabetes, beta cells are in a vicious cycle wherein an impaired insulin response to glucose produces hyperglycaemia, which stresses cell function and limits efficiency [43]. The ageing pancreas and the post-mitotic beta cells within it demonstrate loss of function integrity, which manifests as increased risk for type 2 diabetes. Aged beta cells display upregulated ER stress responses and autophagy [44] in addition to mitochondrial dysfunction and reduced insulin secretion. We now demonstrate these phenomena are effectively reversed by blocking CD47 signalling. However, primary beta cell dysfunction does not wholly explain development of dysglycaemia with age, and islet–endothelial crosstalk provide crucial paracrine signals that enhance survival and function. Further studies have revealed that ageing of islets involves functional abnormalities in endothelial cells, suggesting that age-related impairment of glucose homeostasis is a consequence of vascular ageing [45]. We recently showed that absent CD47 expression was crucial in maintaining vascular function with age and in glucose homeostasis [23]. This suggests that widespread cellular targeting of CD47 signalling would have a beneficial role in preserving the endocrine pancreas and should be tested for senolytic capacity.

The originally ascribed function of CD47 was cognate recognition of self, controlling the ‘don’t-eat-me’ signal through interactions with macrophage-bearing signal regulatory protein α (SIRPα) [46]. This mechanism was subverted by CD47 overexpression, with concurrent inactivation of β2-microglobulin and class II MHC transactivator to generate hypoimmune islets [47, 48]. These cells survived, engrafted and were functional, avoiding allogeneic and autologous immune destruction in immunocompetent diabetic mice [47] and xenogeneic damage in non-human primates [48]. Manipulation of CD47 expression has been used to mitigate beta cell destruction associated with the instant blood-mediated inflammatory reaction (IBMIR) [49]. Given that the immunological and cytoprotective benefits of CD47 on beta cells require diametrically opposed expression levels, the future challenge will be deciding which receptor role to leverage to maximise islet survival and function.

Limitations of this study includes the use of a beta cell line, and the isolation of primary islets only from male mice. Further studies are required to understand variations in plasma TSP1 levels, including sex differences, diurnal variations and the effect of acute variations in blood glucose levels, before we can interrogate whether this protein can serve as a potential biomarker. However, the findings presented here provide robust support for targeting CD47 signal transduction, either via the receptor or the soluble ligand (TSP1), to improve beta cell function and survival and, by extension, glucose control. The current availability of a humanised monoclonal antibody to CD47 further emphasises the substantial translational value of our findings.

## Supplementary Information

Below is the link to the electronic supplementary material.

ESM (PDF 737 KB)

## Data Availability

This manuscript used data acquired from the Human Pancreas Analysis Program (HPAP-RRID:SCR_016202) database (https://hpap.pmacs.upenn.edu/), a Human Islet Research Network (RRID:SCR_014393) consortium (UC4-DK-112217, U01-DK-123594, UC4-DK-112232 and U01-DK-123716).

## Abbreviations

ATG5: Autophagy-related 5
ATG7: Autophagy-related 7
Bcl: B cell lymphoma
BiP: Binding immunoglobulin protein
CHOP: C/EBP homologous protein
ECAR: Extracellular acidification rate
eIF2α: Eukaryotic initiation factor 2α
ER: Endoplasmic reticulum
HIF: Hypoxia inducible factor
IRE1α: Inositol-requiring enzyme 1α
LDH: Lactate dehydrogenase
OCR: Oxygen consumption rate
RT-qPCR: Reverse-transcription quantitative PCR
TSP1: Thrombospondin-1
UPR: Unfolded protein response
WT: Wild-type
