# Supplementary material for: Regulating islet stress responses through CD47 activation
Source: Diabetologia. 2025 Mar 25;68(6):1279–97. doi: 10.1007/s00125-025-06409-3 (PMC12069481; doi:10.1007/s00125-025-06409-3)
Supplement: Supplementary file 1 — ESM (PDF 737 KB) [file 125_2025_6409_MOESM1_ESM.pdf]

ESM Methods

Mouse islet isolation

Mice were sacrificed under isoflurane and their pancreases were perfused with Liberase solution (#5401020001, Sigma-Aldrich, Burlington, MA). After vigorous shaking, the mixture was centrifuged at 350 *g* at 4°C for 3 min and the supernatant was decanted. The pellet was resuspended with M199 media (Sigma Aldrich) and poured through a sieve. The mixture was re-centrifuged, and the supernatant decanted before supplying with cold Ficoll-Paque PLUS density gradient (Sigma-Aldrich) and M199 media without FBS. The mixture was re-centrifuged at 1610 *g* at 4°C for 22 min. The supernatant was collected, and pellet discarded and supplied with M199 media before centrifuging. Islets were allowed to settle by leaving the tube on ice for 4 min and then stained with dithizone dye (Cole-Parmer Scientific Experts, Vernon Hills, IL) to identify beta cells before counting. Islets were hand-picked with a 20 µl pipette under a stereomicroscope for further in-culture experiments prior or embedded into OCT.

Measurement of corrected total cell fluorescence.

At least 3 regions-of-interest were obtained for every slide. Images were analysed for integrated density, area and mean grey value by selecting regions of interest using the selection tool in ImageJ. Background regions (no fluorescence) were also selected and analysed for the same. Size of area selected was kept consistent throughout analyses. From the values obtained, "Corrected Total Cell Fluorescence (CTCF)" was calculated on Excel using the formula: CTCF = Integrated Density – (Area of selected islet × Mean fluorescence of background readings). In brief, for each slide (n = 3) per group, a CTCF average of 3 images was obtained. CTCF immunofluorescence quantification has been published previously[1].

Single Cell RNAseq analysis

The dataset included normalized gene expression matrices and associated cell-type annotations generated by HPAP, encompassing major pancreatic cell populations (e.g., alpha, beta, ductal, endothelial, and immune cells) as previously reported[2-4]. All analyses, including clustering and visualization, were performed in R using the Seurat package unless otherwise indicated.

Quality control (e.g., filtering cells based on gene count or mitochondrial content) was carried out by HPAP, and these parameters were not further modified here. Principal component analysis (PCA) was employed for initial dimensionality reduction, followed by uniform manifold approximation and projection (UMAP) to generate two-dimensional embeddings. Clusters were reviewed to confirm alignment with the original HPAP-provided cell-type labels and not reassigned. Dot plot visualizations were created to illustrate relative gene expression and the proportion of cells in each cluster or sample group.

References

[1] Nourmohammadzadeh M, Xing Y, Lee JW, et al. (2016) A microfluidic array for real-time live-cell imaging of human and rodent pancreatic islets. Lab Chip 16(8): 1466-1472. 10.1039/c5lc01173f

[2] Kaestner KH, Powers AC, Naji A, Consortium H, Atkinson MA (2019) NIH Initiative to Improve Understanding of the Pancreas, Islet, and Autoimmunity in Type 1 Diabetes: The Human Pancreas Analysis Program (HPAP). Diabetes 68(7): 1394-1402. 10.2337/db19-0058

[3] Shapira SN, Naji A, Atkinson MA, Powers AC, Kaestner KH (2022) Understanding islet dysfunction in type 2 diabetes through multidimensional pancreatic phenotyping: The Human Pancreas Analysis Program. Cell Metab 34(12): 1906-1913. 10.1016/j.cmet.2022.09.013

[4] Patil AR, Schug J, Naji A, Kaestner KH, Faryabi RB, Vahedi G (2023) Single-cell expression profiling of islets generated by the Human Pancreas Analysis Program. Nat Metab 5(5): 713-715. 10.1038/s42255-023-00806-x

ESM Table 1: Human pancreas samples

Donor ID	Demographic data<br>Cause of death	Diagnosis
D21-1345<br>Figure 7C	F, 29 y, BMI 37.2<br>Cerebral hypoxia	T1D
D22-0300<br>Figure 4B, 4C, 4D (image and measurements)	F, 55 y, BMI 28.6<br>Intracerebral haemorrhage	T2D
D24-0359<br>Figure 4C, 4D (measurements)	M, 58 y, BMI 28.1<br>Traumatic brain injury	T2D
D22-0665<br>Figure 4B (measurements)	F, 60y, BMI 34<br>Intracerebral haemorrhage	T2D
D22-0554<br>Figure 4B, 4C, 4D (measurements)	M, 59 y, BMI 26.3<br>CVA (MCA infarction)	T2D
D22-0173<br>Figure 4C, 4D (measurements)	F, 19 y, BMI 22.4<br>Traumatic brain injury	Healthy donor, young
D22-0488<br>Figure 4B, 4C, 4D (measurements)	F, 13 y, BMI 19.2<br>Cerebral hypoxia	Healthy donor, young
D22-0485<br>Figure 4B (measurements)	M, 24 y, BMI 25.5<br>Motor vehicle accident	Healthy donor, young
D22-0397<br>Figure 4C, 4D (image and measurements)	M, 7 y, BMI 20<br>Intracerebral haemorrhage	Healthy donor, young
D22-0367<br>Figure 4B (image and measurements), 7C	F, 32 y, BMI 20.3<br>Cerebral hypoxia	Healthy donor, young
D23-0254<br>Figure 4B, 4D (measurements)	M, 70 y, BMI 29.3<br>Intracerebral haemorrhage	Healthy donor, aged
D22-0871<br>Figure 4B (measurements)	F, 65 y, BMI 23.5<br>Cardiac arrest, cerebral hypoxia	Healthy donor, aged
D24-0645	M, 63 y, BMI 26	Healthy donor, aged

Figure 4B (image and measurements)	Cardiac arrest, cerebral hypoxia	
D22-1431<br>Figure 4C (measurements)	M, 69 y, BMI 26.5<br>Intracerebral haemorrhage	Healthy donor, aged
D23-0607<br>Figure 4C, 4D (image and measurements)	M, 76 y, BMI 26.8<br>Traumatic brain injury	Healthy donor, aged
D23-1379<br>Figure 4C, 4D (measurements)	F, 79 y, BMI 36.9<br>Out of hospital cardiac arrest	Healthy donor, aged
D22-0871<br>Figure 4B (measurements)	F, 64 y, BMI 23.5<br>Out of hospital cardiac arrest, cerebral hypoxia	Healthy donor, aged

ESM Table 2: Primary antibodies

Antibody	Target	Dilution	Molecular Weight of protein (kDa)	Catalogue #	Source
Mouse α-CD47<br>(B6H12)	Human CD47	SDS-PAGE (in blocking buffer): 1.5:1000<br>Blocking CD47 in cells/islets/tissue: 1μg/ml<br>Immunofluorescence (in 2% BSA): 1:150	~50	sc-12730	Santa Cruz Biotechnology, Dallas, TX
Rat α-CD47<br>(MIAP301)	Mouse CD47	SDS-PAGE (in blocking buffer): 1.5:1000<br>Blocking CD47 in cells/islets/tissue: 1μg/ml<br>Immunofluorescence (in 2% BSA): 1:150 (tissues), 1:50 (cells)	~50	sc-12731	Santa Cruz Biotechnology
Mouse α-Insulin (L6B10)	Mouse and Human	SDS-PAGE (in blocking buffer): 1:1000<br>Immunofluorescence (in 2% BSA): 1:150 (tissues)	~6	8138S	Cell Signalling Technology, Danvers, MA
Rabbit α-Insulin<br>(C27C9)	Mouse and Human	SDS-PAGE (in blocking buffer): 1:1000<br>Immunofluorescence (in 2% BSA): 1:150 (tissues) or 1:50 (cells)	~6	3014S	Cell Signalling Technology
Rabbit α-TSP1	Human and mouse TSP1	SDS-PAGE (in blocking buffer): 1:1000	~155	ab85762	Abcam, Cambridge, UK

Mouse α-TSP1	Human TSP1	Immunofluorescence (in 2% BSA): 1:150 (tissues)	–	ab1823	Abcam
Rabbit α-HIF-1α (EPR16897)	Human and mouse HIF-1α	SDS-PAGE (in blocking buffer): 1:1000	~110	ab179483	Cell Signalling Technology
Rabbit α-Bcl-2 (D17C4)	Human and mouse Bcl-2	SDS-PAGE (in blocking buffer): 1.5:1000	~26	3498S	Cell Signalling Technology
Rabbit α-Bcl-XL (54H6)	Human and mouse Bcl-XL	SDS-PAGE (in blocking buffer): 1.5:1000	~30	2764S	Cell Signalling Technology
Rabbit α-Vinculin (E1E9V) XP®	Mouse and Human Vinculin	SDS-PAGE (in blocking buffer): 1:1000	~124	13901S	Cell Signalling Technology
Rabbit α-BiP	Mouse and Human BiP	SDS-PAGE (in blocking buffer): 1:1000	~78	3177S	Cell Signalling Technology
Rabbit α-phospho-(p)- eIF2α (Ser51)	Mouse and Human p-eIF2α	SDS-PAGE (in blocking buffer): 1:1000	~38	9721S	Cell Signalling Technology
Rabbit α-total eIF2α	Mouse and Human total-eIF2α	SDS-PAGE (in blocking buffer): 1:1000	~38	9722S	Cell Signalling Technology
Rabbit α-IRE1α (14C10)	Mouse and Human IRE1α	SDS-PAGE (in blocking buffer): 1:1000	~130	3294S	Cell Signalling Technology
Rabbit α-CHOP	Mouse CHOP	SDS-PAGE (in blocking buffer): 1:1000	~27	5554S	Cell Signalling Technology
Rabbit α-Atg5 (D5F5U)	Mouse/human Atg5	SDS-PAGE (in blocking buffer): 1:1000	~55	12994S	Cell Signalling Technology
Rabbit α-Atg7 (D12B11)	Mouse/human Atg7	SDS-PAGE (in blocking buffer): 1:1000	~78	8558S	Cell Signalling Technology

Rabbit α-Beclin-1 (D40C5)	Mouse/human Beclin-1	SDS-PAGE (in blocking buffer): 1:1000	~60	3495S	Cell Signalling Technology
Rabbit α-p62	Mouse/human p62	SDS-PAGE (in blocking buffer): 1:1000	~60	ab91526	Abcam
Rabbit LC3A/B (D3U4C)	Mouse/human LC3 I/II	SDS-PAGE (in blocking buffer): 1:1000	I ~14<br>II ~16	12741S	Cell Signalling Technology
Mouse α-β-actin (8H10D10)	Mouse/human β-actin	SDS-PAGE (in blocking buffer): 1:2500	~42	3700S	Cell Signalling Technology
Rabbit α-p16-INK4A	Human $p16^{INK4A}$	Immunofluorescence (in 2% BSA): 1:100	–	10883-1-AP	Proteintech, Rosemont, IL
Rabbit α-p21 (EPR362)	Human $p21^{cip1}$	Immunofluorescence (in 2% BSA): 1:100	–	ab188224	Abcam
Rat α-IgG2a (RMG2a-62) Isotype Control	Mouse IgG	Immunofluorescence (in 2% BSA): 1:150 (tissues) or 1:50 (cells)	–	sc-53758	Santa Cruz Biotechnology
Mouse α-IgG Antibody (D-1) Isotype Control	Human IgG	Immunofluorescence (in 2% BSA): 1:150 (tissues) or 1:50 (cells)	–	sc-515946	Santa Cruz Biotechnology

ESM Table 3: Secondary Antibodies

Antibody	Dilution	Source
Alexa Fluor 488 | 546 α-Mouse<br>Alexa Fluor 488 | 546 α-Rabbit<br>Alexa Fluor 488 | 546 α-Rat	For immunofluorescence: 1:100 (in PBS-T)	Invitrogen, Waltham, MA
Li-COR<br>Green | Red α-Mouse<br>Green | Red α-Rat<br>Green | Red α-Rabbit	For SDS-PAGE: 1:10000 (in TBS-T)	Li-COR Biosciences, Lincoln, NE

ESM Table 4: Taqman Primers (ThermoFisher Scientific)

Gene	Assay ID
Rn18S	Mm03928990_g1
Thbs1	Mm00449032_g1
CD47	Mm00495011_m1
Atg5	Mm01187303_m1
Atg7	Mm00512209_m1
Beclin-1	Mm01265461_m1
Sqstm1 (p62)	Mm0448091_m1
Pou5f1 (Oct3/4)	Mm03053917_g1
Sox2	Mm03053810_s1
Klf4	Mm00516194_m1
Myc	Mm00487804_m1
Hspa5 (BiP)	Mm00517691_m1
ERN1 (IRE1α)	Mm00470233_m1
Ddit3 (CHOP)	Mm07294308_m1

ESM Figure 1

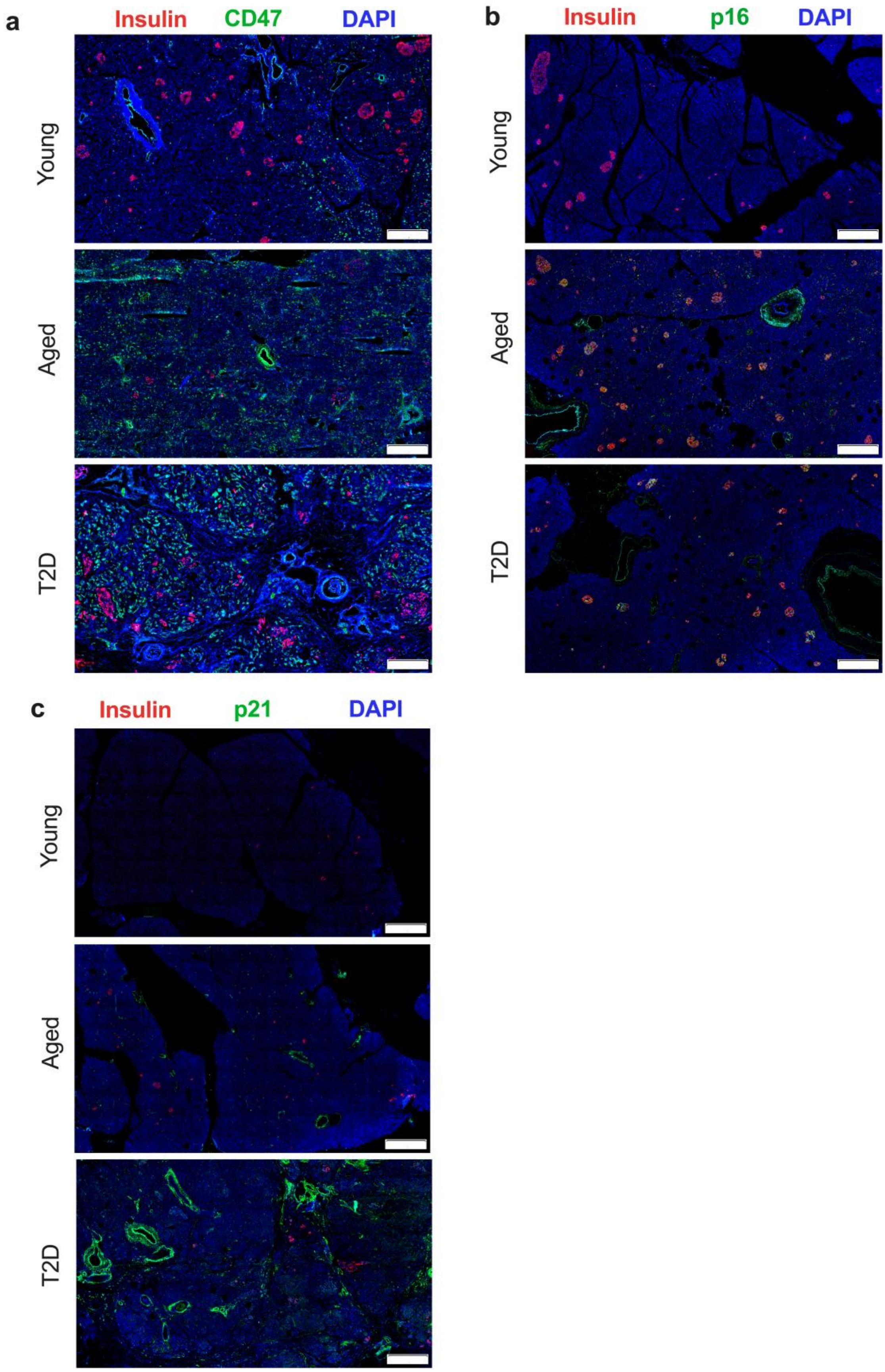
a
Insulin CD47 DAPI
Young
Aged
T2D
b
Insulin p16 DAPI
Young
Aged
T2D
c
Insulin p21 DAPI
Young
Aged
T2D

Senescence is upregulated by ageing and diabetes. Human pancreas samples from non-diabetic young (n=5) and aged (n=6) organ donors, as well as donors with T2D (n=4) were stained for (a) insulin and CD47, (b) insulin and p16, as well as (c) insulin and p21. Images were obtained at 4x magnification. Scale bar: 500 µm.

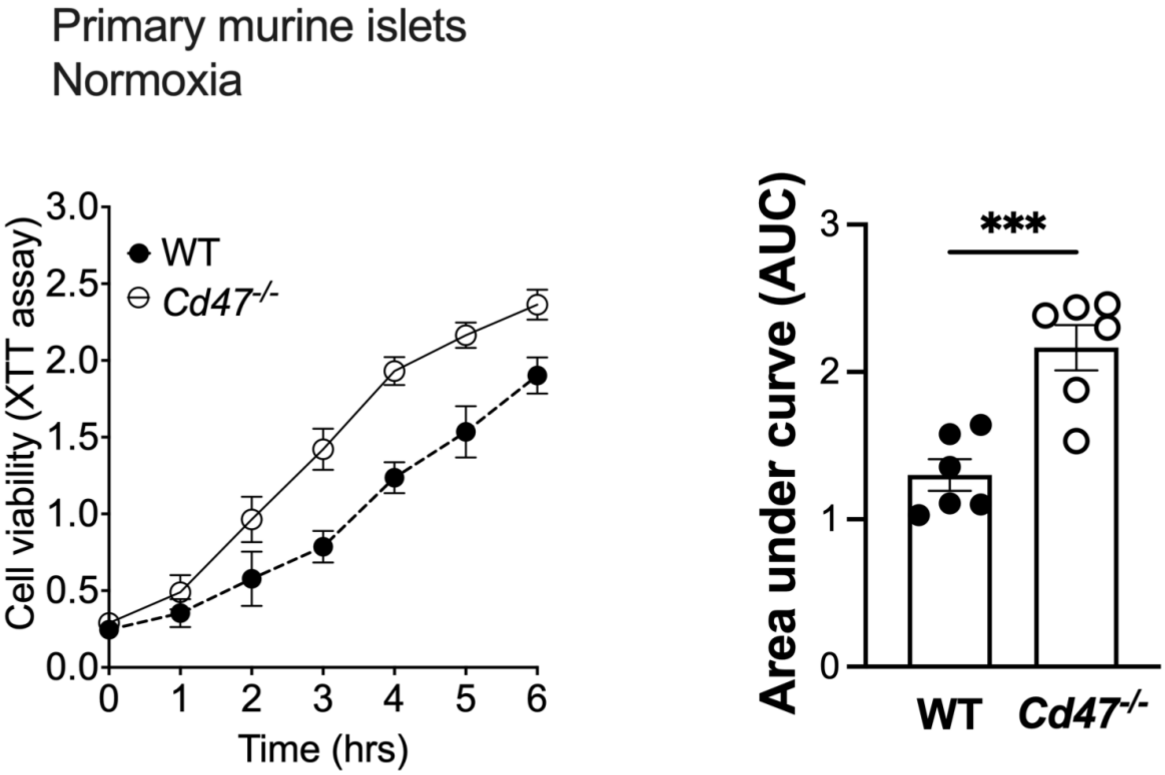

ESM Figure 2

CD47 signalling limits beta cell viability. WT and $CD47^{-/-}$ murine islets (from n=3-5 samples isolated from n=3-4 mice/sample) were isolated and XTT viability assay was performed under standard culture conditions.

Checklist for reporting human islet preparations used in research

Adapted from Hart NJ, Powers AC (2018) Progress, challenges, and suggestions for using human islets to understand islet biology and human diabetes. Diabetologia https://doi.org/10.1007/s00125-018-4772-2

Islet preparation	1	2	3	4	5	6	7	8[a]
MANDATORY INFORMATION								
Unique identifier	H250 (D17-0003	H259 (D17-0314)	H263 (D17-0556)	H265 (D18-0065)	H298 (D21-0422)	H300 (D21-0510)	H304 (D22-1103)	H299 (D21-0448)
Donor age (years)	44	55	47	57	47	48	44	28
Donor sex (M/F)	M	F	M	F	F	F	M	M
Donor BMI (kg/m^2)	30.86	20.24	27.68	23.31	44.41	23.44	24.4	30.19
Donor HbA_{1c} or other measure of blood glucose control	No insulin requirement, random BGL 8mmol/L	No insulin requirement, random BGL 8.2mmol/L	No insulin requirements, BGL 9.7mmol/L	No insulin requirements, BGL 7.3mmol/L	No insulin requirement, BGL<10mmol/L	No insulin requirement, random BGL 6.8mmol/L	No insulin requirement, random BGL 4.8mmol/L	No insulin requirement, BGL 16.3
Origin/source of islets[b]	NSW	NSW	National Islet Transplant Consortium Australia	National Islet Transplant Consortium Australia	National Islet Transplant Consortium Australia	NSW	VIC	National Islet Transplant Consortium Australia
Islet isolation centre	Westmead Hospital	Westmead Hospital	Westmead Hospital	Westmead Hospital	Westmead Hospital	Westmead Hospital	Westmead Hospital	Westmead Hospital
Donor history of diabetes? Please select yes/no from drop down list	No	No	No	No	No	No	No	No
**If Yes, complete the next two lines if this information is available**								

Diabetes duration (years)								
Glucose-lowering therapy at time of death[c]								
**RECOMMENDED INFORMATION**								
Donor cause of death	Hypoxic brain injury	Subarachnoid hemorrhage	Intracranial hemorrhage	Cerebral hypoxia	Cerebral hypoxia	Intracranial hemorrhage	Cerebral infarct	Traumatic brain injury
Warm ischaemia time (h)	Not available	Not available	Not available	Not available	Not available	Not available	Not available	
Cold ischaemia time (h)	14.6	12.83	8.7	24.7	26.2	11.54	20.2	20.1
Estimated purity (%)	75%	60%	60%	80%	80%	70%	70%	50%
Estimated viability (%)	82.6%	95%	100%	95%	80%	90%	81.5%	80%
Total culture time (h)[d]	<24 h	<24 h	<24 h	<24 h	<24 h	<24 h	<24 h	<24 h
Glucose-stimulated insulin secretion or other functional measurement[e]	Stimulation index 1.15	Stimulation index 2.12	Stimulation index 1.01	Stimulation index 1.17	Not performed	Not performed	Not performed	Not performed
Handpicked to purity? Please select yes/no from drop down list	No	No	No	No	No	No	No	No
Additional notes	Used in Figures 1A, 1B	Used in Figures 1A, 1B	Used in Figures 1A, 1B	Used in Figure 1B	Used in Figures 1A, 1C, 1D, 5F, 7A, 7B	Used in Figures 1A, 1C, 1D, 5F	Used in Figures 1A, 1C, 1D, 5F, 7A, 7B	Used in Figures 1C, 1D, 5F, 7A, 7B

[a]If you have used more than eight islet preparations, please complete additional forms as necessary

[b]For example, IIDP, ECIT, Alberta IsletCore

[c]Please specify the therapy/therapies

[d]Time of islet culture at the isolation centre, during shipment and at the receiving laboratory

[e]Please specify the test and the results

Islet preparation	1	2	3	4	5	6	7	8[a]
**MANDATORY INFORMATION**								
Unique identifier	H306 (D23-0329)	H302 (D21-0956)	H281	H297				
Donor age (years)	46	33	56	60				
Donor sex (M/F)	F	F	F	M				
Donor BMI (kg/m^2)	25.5	46.4	27.34	25.1				
Donor HbA_{1c} or other measure of blood glucose control	No insulin requirement, random BGL 8.9mmol/L	Not recorded	Not recorded	No insulin requirement, random BGL 8.6				
Origin/source of islets[b]	NSW	National Islet Transplant Consortium Australia	National Islet Transplant Consortium Australia	National Islet Transplant Consortium Australia				
Islet isolation centre	Westmead Hospital	Westmead Hospital	Westmead Hospital	Westmead Hospital				
Donor history of diabetes? Please select yes/no from drop down list	No	No	No	No				
**If Yes, complete the next two lines if this information is available**								
Diabetes duration (years)								
Glucose-lowering therapy at time of death[c]								

RECOMMENDED INFORMATION								
Donor cause of death	Intracranial hemorrhage	Cerebral ischemia	MVA – traumatic brain injury	Hemorrhagic cerebrovascular accident				
Warm ischaemia time (h)	Not available	Not available	Not available	Not available				
Cold ischaemia time (h)	14.5	16.8	5.85	5.18				
Estimated purity (%)	70%	40%	80%	25%				
Estimated viability (%)	100%	90%	80%	95%				
Total culture time (h)[d]	<24 h	<24 h	<24 h	<24 h				
Glucose-stimulated insulin secretion or other functional measurement[e]	Not performed	Not performed	Not performed	Not performed				
Handpicked to purity? Please select yes/no from drop down list	No	No	No	No				
Additional notes	Used in Figures 1C, 1D	Used in Figures 1C, 1D	Used in Figure 5F	Used in Figure 5F				
